# A plasmon modulator by directly controlling the couple of photon and electron

**DOI:** 10.1038/s41598-022-09176-y

**Published:** 2022-03-28

**Authors:** Xue-fang Hu, Xiang-yue Zhao, Yin-wei Gu, Shu-ping Jin, Yi-ping Cui, Chang-gui Lu

**Affiliations:** Advanced Photonics Center, Southeast University, Nanjing, 210096 China

**Keywords:** Electronic properties and devices, Graphene

## Abstract

The manipulation of surface plasmon polaritons plays a pivotal role in plasmonic science and technology, however, the modulation efficiency of the traditional method suffers from the weak light-matter interaction. Herein, we propose a new method to overcome this obstacle by directly controlling the couple of photon and electron. In this paper, a hybrid graphene-dielectric- interdigital electrode structure is numerically and experimentally investigated. The plasmon is excited due to the confined carrier which is regulated by the potential wells. The frequency of plasmon can be tuned over a range of ~ 33 cm^−1^, and the obtained maximum extinction ratio is 8% via changing the confined area and the density of carrier. These findings may open up a new path to design the high efficiency all-optical modulator because the electrons can also be driven optically.

## Introduction

The optical modulator can control the polarization, frequency, amplitude and phase of light, which plays a vital role in the high-speed communication systems^1–3^. The traditional optical modulator is mainly based on electro-optic crystal, semiconductor, microring resonator, Mach–Zehnder interferometer (MZI) and photonic crystal^4–9^. For example, the group of Kevin F. MacDonald demonstrated an all-optical modulator in which the propagating surface plasmon polaritons (SPPs) was controlled by the pump light via interacting with the aluminum interface^10^. The group of S. Sederberg proposed an ultrafast all-optical switching in a silicon-based plasmonic nanoring resonator. The transmitted signal is controlled by the photo-generated free carriers which changes the complex refractive index of the nanoring resonator^11^. The group of Yanjun Bao presented a graphene device to modulate the propagation of SPPs, the source/drain electrode of this device was made of a reflective antenna pair structure whose optical resonance was tuned by the applied voltage^12^. However, most of them modulate the light through controlling the optical properties of the medium, and then the modulation performances depend on the characteristics of medium greatly. Thus, the modulation efficiency is relatively low suffered from the weak light-matter interaction.

SPPs are modes of electromagnetic waves propagating along a metal surface by the interaction of light waves and surface charges. In this mode, the light wave is localized at the interface between metal and dielectric, and there is a strong coupling between the light field and the free electrons on the metal surface, which offers a unique way to concentrate optical fields down to nanometer-sized regions^13–17^. Various plasmonic optical components, including lenses, reflectors, and waveguides, have been reported, demonstrating the potential capabilities of plasmonics to shrink the photonic circuits below the diffraction limit^18–22^. However, the modulation efficiency of the traditional method suffers from the weak light-matter interaction. Fortunately, the movement of electrons can affect the behaviors of SPPs effectively, a method that can directly control the characteristics (such as the movement and density) of electrons is desired to be proposed to improve the modulation efficiency. In addition, it is difficult to modulate the concentration of electron in metal while it is feasible in some semiconductor and two-dimensional materials^23–26^. Graphene is a promising material in photonic and optoelectronic applications due to its superiority of high carrier mobility, broadband optical response and facile electrical tunability^27–31^. It is reported that highly-doped graphene can support SPPs with stronger mode confinement and lower propagation losses compared to the noble metal. Benefiting from these unique electronic characteristics, graphene has become a viable foundation for developing highly integrated plasmonic devices and has a potential application in photodetector, modulator, polarizer and biosensor^32–34^.

In this letter, we propose a graphene plasmon modulator consisting of a hybrid graphene-dielectric-interdigital electrode structure. The movement of carrier in graphene can be directly regulated in the potential wells formed by the interdigital electrode, and then the SPPs can be excited. The frequency of plasmon can be tuned over a range of ~ 33 cm^−1^ and the obtained maximum extinction ratio is 8% by changing the confined area and the density of carrier. (The group of Weilu Gao^42^ proposed a grating compensation method for the excitation of SPPs and achieved a ER of 6%, the modulation depth is improved by our method). This method may pave a novel way for the high efficiency all-optical modulator because the electrons can also be driven optically. Compared with the traditional plasmon modulator, the performance of proposed modulator is independent of medium, and the couple of photon and electron is directly controlled. It will have a promising application in optical communication, integrated sensor and photonic circuits^35,36^.

## Model analysis

**Figure 1:**
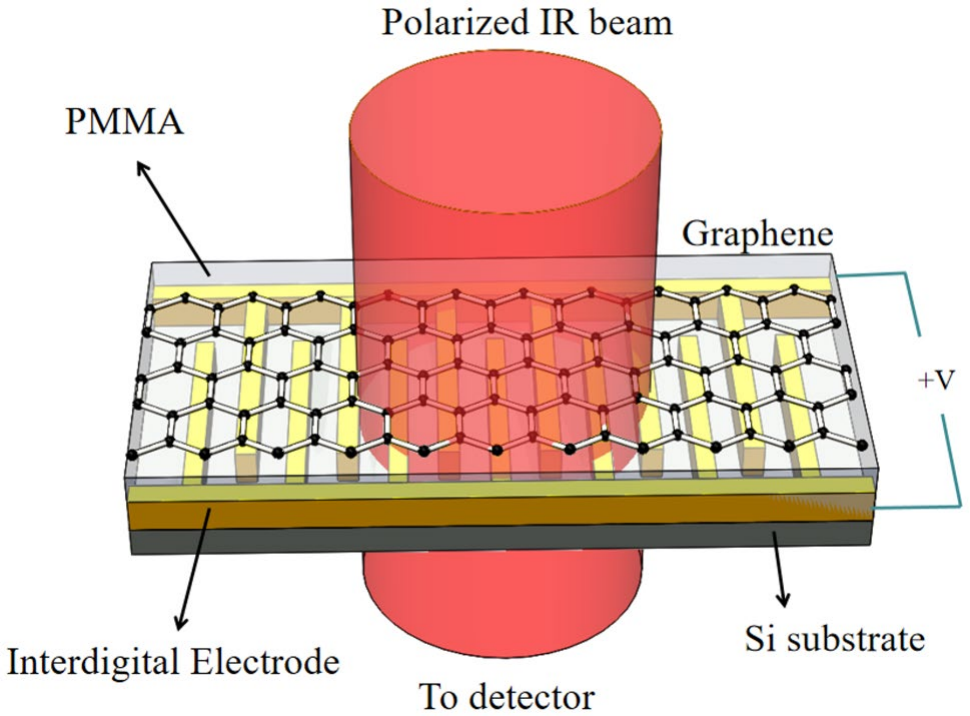
The 3D schematic of the modulator, the movement and density of carrier are controlled by the voltage applied on the interdigital electrode. The energy of excited SPPs will be absorbed in the graphene layer and result in a resonant dip in transmission, which can be collected by the detector.

Figure 1 illustrates the three dimensional (3D) schematic of the modulator which consists of a hybrid graphene-dielectric-interdigital electrode structure. The interdigital electrode is designed to control the movement of carrier in graphene. The polymethyl methacrylate (PMMA) acts as the dielectric layer and the monolayer graphene is the transmission medium of SPPs. The carrier in graphene will reflect when its energy is smaller than the potential well established by the interdigital electrode, thus, the SPPs can be excited owing to the confined carrier in the potential well^37^. The energy of SPPs excited by the polarized infrared (IR) light will be absorbed in the graphene layer, which may introduce a resonant dip in the transmission and can be collected by the detector. By means of applying different voltage on the interdigital electrode, the frequency of plasmon can be controlled through changing the confined area and the density of carrier.

To understand the relationship between the resonance frequency and the changes of carrier in our model, the parameters which can affect the resonant frequency of graphene nano-ribbons are investigated. Under a normal-incidence beam, the resonance frequency *ω* of the graphene nano-ribbons can be expressed as follows^38^:

1 $$ \omega = \sqrt {\frac{{2\pi e^{2} E_{f} }}{{\varepsilon_{0} \hbar^{2} d}} \circ } $$

2 $$ E_{f} = \hbar v_{f} \sqrt {\pi \left| n \right|} $$

where $\hbar$, e and ɛ_0_ are the reduced Planck constant, the charge of an electron and the permittivity of vacuum respectively, and *E*_*f*_ represents the Fermi energy. It can be obtained from Eqs. (1) and (2) that the resonance frequency of the graphene nano-ribbons *ω* varies with the width *d* and carrier density *n* (*d*^−1/2^ and *n*^1/4^). The voltage applied on the interdigital electrode can introduce a potential well to control the movement of carrier and only the carrier perpendicular to the interdigital electrode is modulated. It is like a nano-ribbons if the carrier concentrates on the center of graphene and no carrier locates on the boundary. In this condition, the confined area is similar to the width of nano-ribbons^39–41^. Thus, the Eqs. (1) and (2) could also give the theoretical prediction of the resonance frequency versus the changes of carrier in the proposed model. The resonance frequency is varied with the confined area and the density of carrier.

## Results and discussion

**Figure 2:**
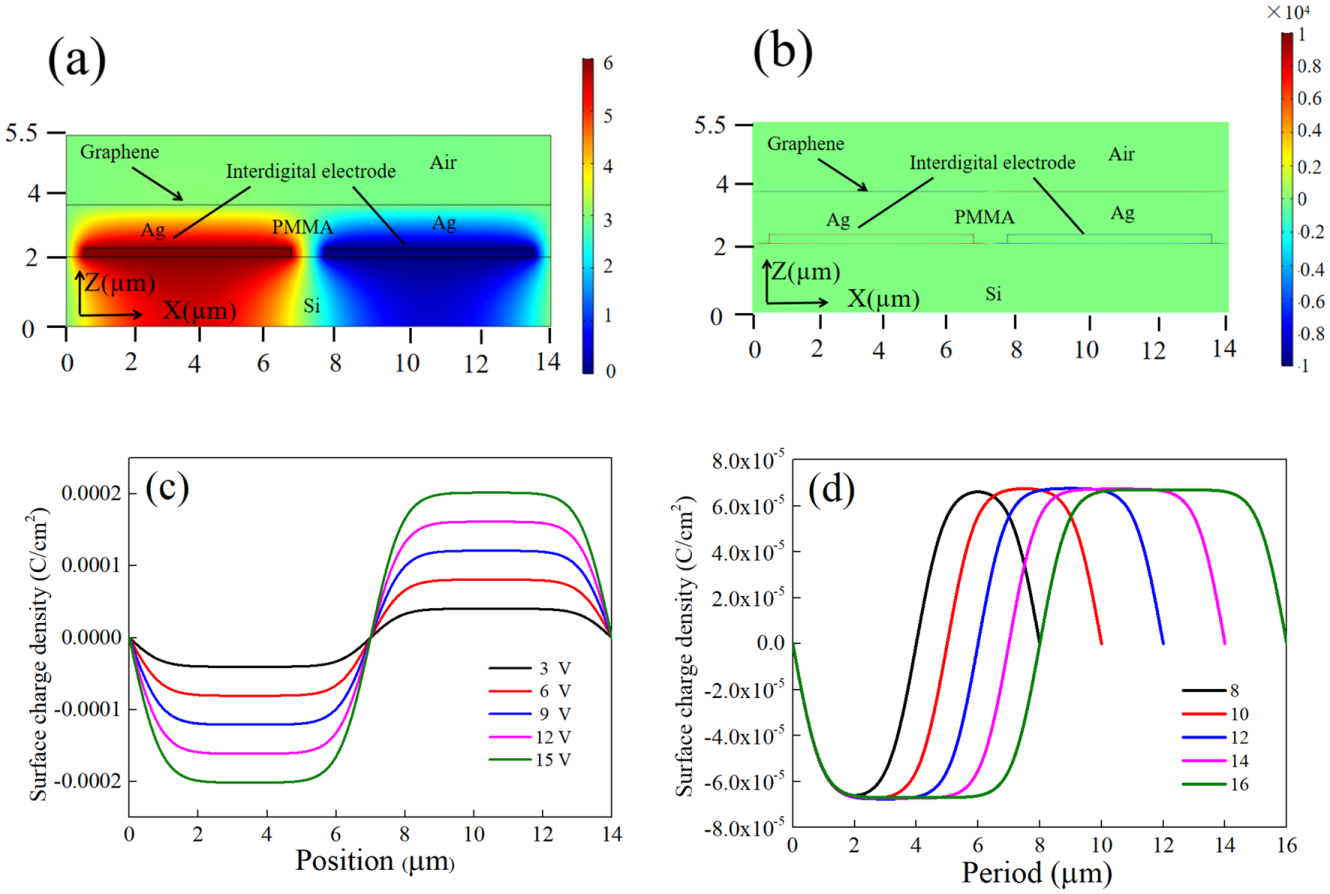
(**a**) The electrical potential of the proposed structure (**b**) surface charge density distribution on the graphene under the voltage of 6 V (**c**) the surface charge density distribution versus different voltage (**d**) the surface charge density distribution versus different period of electrode.

Under the voltage of 6 V, we analyze the electrical potential and the surface charge density distribution of the proposed model in Fig. 2a, b. It can be seen that the charge distributes on the surface of graphene periodically when the voltage is applied and it can also be regard as the carrier distribution because the surface charge is originate from the carrier in graphene. Figure 2c shows the distribution of surface charge versus the position under different voltage. It can be seen that the electrons are limited in a narrower area with a higher density when the voltage is increased. Figure 2d shows the surface charge distribution versus different periods of interdigital electrodes. It demonstrated that a longer period corresponds to a wider confined area of carrier at the same voltage. As the important factors that affect the plasmon frequency, both the confined area and the density of the carrier in graphene can be used to control the resonance frequency effectively by means of altering voltage or changing the period of interdigital electrode.

**Figure 3:**
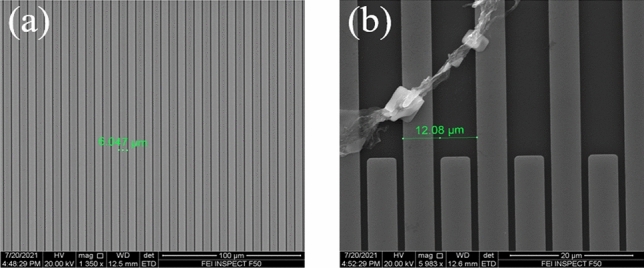
The SEM image of the sample (**a**) in the center (**b**) at the bondary of interdigital electrode.

The processes of fabricating the proposed structure mainly contains following three steps: (1) Traditional photolithography and metal lift-off techniques are used to fabricate the interdigital electrodes on the silicon substrate. The linewidth, period and thickness of interdigital electrodes are 5 μm, 12 μm and 80 nm respectively. (2) A PMMA layer (with a thickness of 100 nm) is then spin on the top of interdigital electrodes. (3) A monolayer graphene is transferred on the PMMA layer by means of wet-transfer techniques^42^. The quality of prepared interdigital electrodes and graphene are demonstrated by the scanning electron microscope(SEM). The SEM images of the sample in the center and bondary of interdigital electrode are shown in Fig. 3a, b respectively. As the monolayer graphene is transparent and well transferred in the center of the interdigital electrode, we can not distinguish whether it exist from Fig. 3a. The image, which exist some cracking at the boundary of interdigital electrode, is selected to verified the existence of graphene as shown in Fig. 3b. The influence of cracking graphene on the transmission spectra can be ignored because the incident light is mainly focused on the center of electrode and its spot size is about 500 × 500 μm, just a half size of the interdigital electrode.

**Figure 4:**
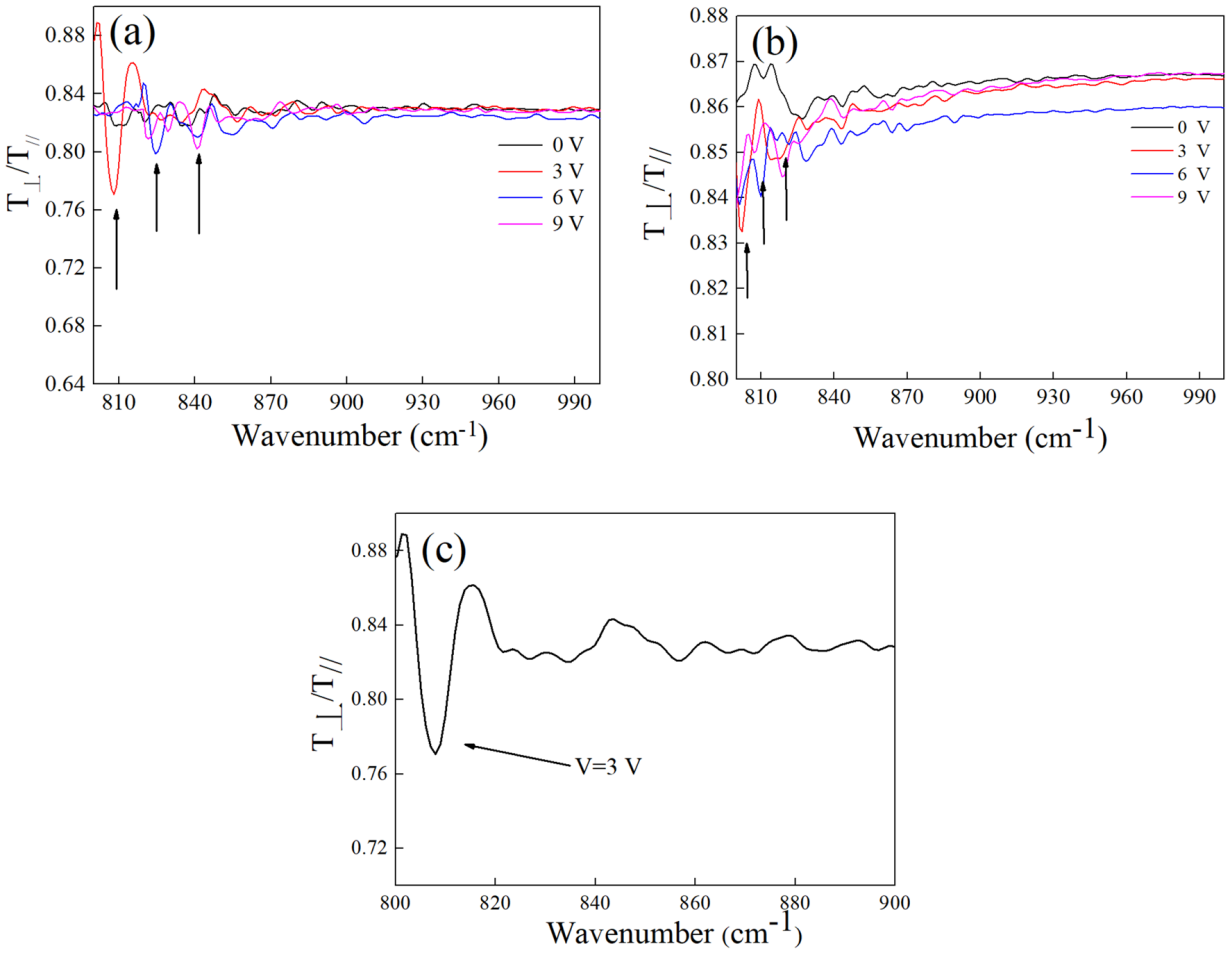
The transmission spectra at different voltage of the sample (**a**) with a period of 12 um (**b**) with a period of 14 um (**c**) the maximum ER of the sample is about 8%.

We performed polarization-dependent transmission experiments on the fabricated sample using Fourier transform infrared spectroscopy (FTIR) in the mid-infrared region (Bruker,Vertex 80 V). The polarization of the light is controlled by a MgF_2_ polarizer and the light is focused on the center of interdigital electrode by a lens. The SPPs can be excited only when the incident light is polarized perpendicular to the interdigital electrode. Its energy will be absorbed in the graphene layer and introduces a resonant dip in the transmission spectrum (${\text{T}}_{ \bot }$). No resonance features are observed on the transmission spectrum when the incident light is polarized parallel to the interdigital electrode (${\text{T}}_{\parallel }$). Here, the transmission spectra (${{{\text{T}}_{ \bot } } \mathord{\left/ {\vphantom {{{\text{T}}_{ \bot } } {{\text{T}}_{\parallel } }}} \right. \kern-\nulldelimiterspace} {{\text{T}}_{\parallel } }}$) of two samples with different period are plotted in Fig. 4a, b respectively. The transmission spectrum of the sample with a linewidth of 5 μm and period of 12 μm is shown in Fig. 4a. A blue shift of 33 cm^−1^ in resonance frequency is obtained (807 cm^−1^, 825 cm^−1^, 840 cm^−1^) as the voltage increases from 3 to 9 V. It is caused by the increase of carrier density and the minimization of confined area at the same time. The transmission spectrum is smooth when the voltage is 0 V because the plasmon can not be excited in this condition. The small fluctuation appeared in the spectra is caused by the instrument and the influence on the experiment can be neglected as the amplitude of resonant dip is larger than fluctuation greatly. The amplitude of the resonant dip depends greatly on the number of carrier which could resonate with the incident light. The resonant dip is maximum at a relative low voltage which is because the carrier is confined in a narrower area when the voltage increases and thus some carrier on the boundary can not interact with the incident light. The transmission spectra of the sample with a linewidth of 5 μm and period of 14 μm are shown in Fig. 4b. Compared with Fig. 4a, the resonant dip is located at a lower frequency and be of a smaller shift owing to the wider confined area under the same voltage, which matches well with the numerical results. The maximum extinction ratio (ER) of the proposed structure is approximately 8%, shown in Fig. 4c. The ER can be optimized by improving the quality of graphene and interdigital electrode.

**Figure 5:**
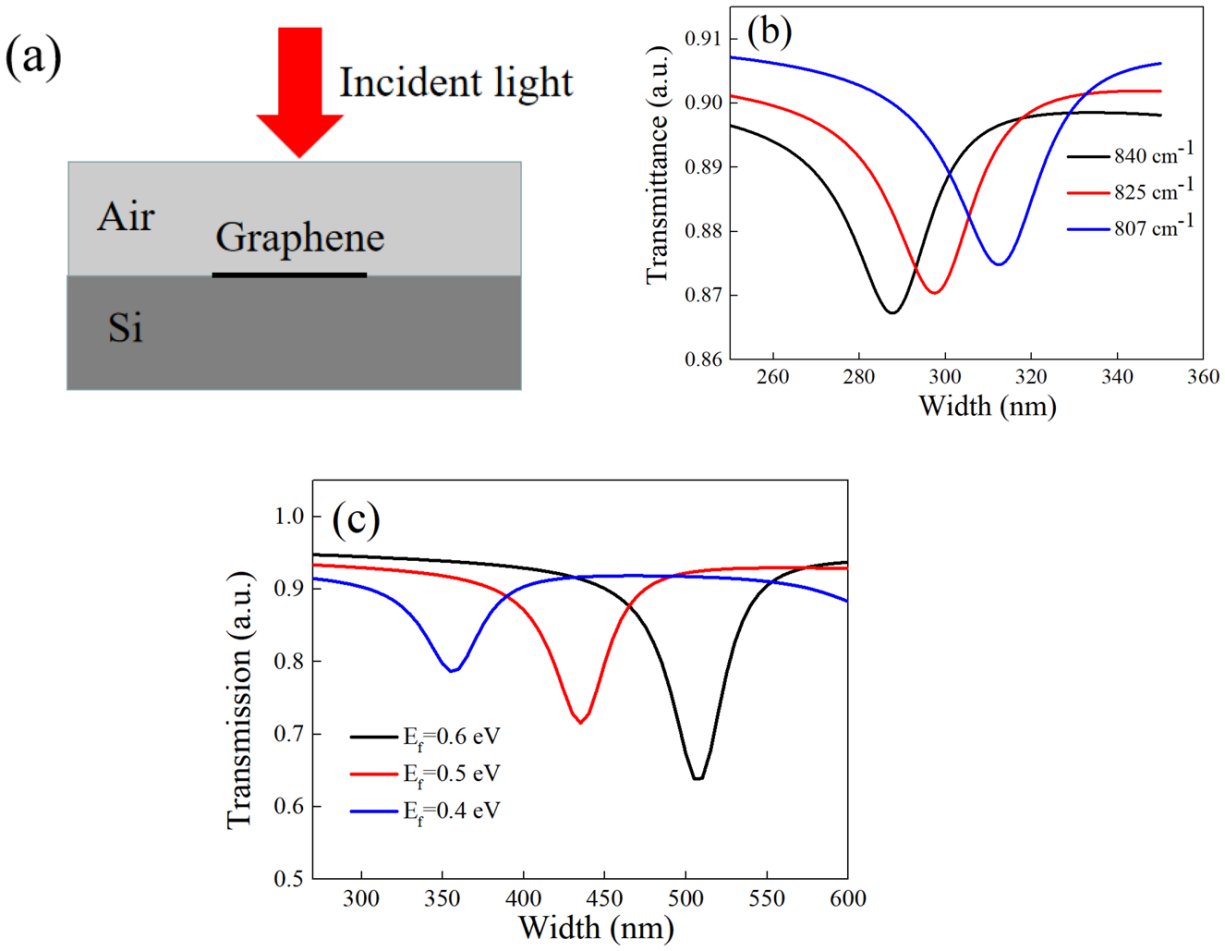
(**a**) The schematic of simulation. (**b**) The transmission versus the width at the frequency of 840, 825 and 807 cm^−1^ respectively (**c**). The transmission versus the width at different Fermi energy.

To verify the influence of confined area and increased carrier density on the resonance frequency, the transmission spectrum of graphene nano-ribbon with different width and carrier density is simulated. Figure 5a is the schematic of the model utilized for simulation and the transmission spectrum as a function of width at different frequencies is shown in Fig. 5b. It can be observed that an obvious resonant dip has appeared at the widths of 289 nm, 299 nm and 314 nm when the wavenumber of the incident light are 840, 825 and 807 cm^−1^ respectively, which demonstrates that a narrower confined area will lead to the blue shift of the frequency. The transmission spectrum versus the width at different Fermi level is also shown in Fig. 5c, it is clear that the increased Fermi level (caused by the increased carrier density) can make the transmission dip move to a longer width, which means the influence of increased carrier density is equivalent with the graphene nano-ribbons with a narrower width. Specifically, the increased carrier density also lead to the blue shift of frequency. Additionally, the amplitude of transmission dip is increased as the carrier density increased. It is because the number of carrier resonate with the incident light increased, which is consistent with the analysis in spectra measurement. In our experiment, the blue shift of frequency is caused by both the narrowed confined area and increased density of carrier at the same time. Although it is hard to predict the accurate changes in confined area and the carrier density, the tendency in experiment is consistent with the theoretical prediction and the numerical result.

## Conclusion

In conclusion, we propose a new type of graphene plasmon modulator by directly controlling the couple of photon and electron. The modulator is demonstrated by a hybrid graphene-dielectric-interdigital electrode structure. The confinement of the carrier can be dynamically controlled by the potential well established from interdigital electrode and the plasmon can be excited. The frequency of plasmon can also be tuned by changing the confined area and the density of carrier. The experimental results match well with the theoretical predictions and the simulations. Additionally, our proposed modulator opens up a new direction for high efficiency all-optical modulators.

## Methods

The simulation is performed using the commercial finite element method (FEM), trial version of software “COMSOL Multiphysics 5.5″. The module of electrostatic is used to obtain the surface charge distribution of the structure, and the module of radio frequency is used to verify the relationship between resonance frequency and changes in carrier. The surface conductivity model and the transitional boundary condition are used in the model of graphene, and the details are discussed in the references^43^.

The interdigital electrode is fabricated using the photolithography and metal lift-off techniques on a silicon substrate with a size of 5 × 8 × 0.52 mm. The number of the electrode is 200, and the adhesion layer of chrome is 30 nm. The thickness of the gold electrode is 100 nm. The PMMA is span on the fabricated electrode with a thickness about 100 nm. The graphene layer is grown by chemical vapor deposition (CVD) on a copper foil and then transferred using PMMA assisted wet-transfer techniques. Before the transfer process, a PMMA layer is spin-coated on graphene on the copper foil, and the copper foil is then etched away in a ammonium persulfate solution bath for about six hours. The PMMA-graphene film floating on the etchant is moved to distilled water several times to rinse the etchant residue and then scooped by the sample patterned with interdigital electrode. Finally, the fabricated sample is dried on the heating stage for half an hour with a temperature of 120 °C.

The measurement about the transmission is using Fourier transform infrared spectroscopy (FTIR) in the mid-infrared region (Bruker, Vertex 80 V). The polarization of the light is controlled by a MgF_2_ polarizer and the light is focused on the center of interdigital electrode by a lens with a size about 500 × 500 μm.

### Ethical approval

This article does not present research with ethical considerations.

## Data Availability

The data relative to the experiments discussed in this work are available upon reasonable request from the corresponding author Changgui Lu.
